# Comparison of proximal femoral universal nail and proximal femoral nail anti-rotation internal fixation for older patients with intertrochanteric femoral fracture: a retrospective cohort study

**DOI:** 10.3389/fmed.2025.1642909

**Published:** 2025-08-11

**Authors:** Yuan Cao, Jixing Fan, Zengzhen Cui, Youliang Hao, Shengtao Lai, Yun Tian, Yang Lv, Fang Zhou

**Affiliations:** ^1^Department of Orthopaedics, Peking University Third Hospital, Beijing, China; ^2^Engineering Research Center of Bone and Joint Precision Medicine, Ministry of Education, Beijing, China

**Keywords:** intertrochanteric femoral fractures, proximal femoral universal nail, proximal femoral nail anti-rotation, fixation failure, medial and lateral wall fractures

## Abstract

**Introduction:**

Intertrochanteric femoral fractures (IFF) are among the most common lower limb fractures in the older population. Our team has developed a new cephalomedullary nail design called the proximal femoral universal nail (PFUN). This study aimed to compare the clinical outcomes of PFUN and proximal femoral nail anti-rotation (PFNA) internal fixation for the treatment of IFF in older patients.

**Methods:**

We retrospectively reviewed the clinical records of 435 older patients with IFF who were treated at Peking University Third Hospital between January 2022 and January 2024. Among them, 215 underwent PFUN (PFUN group) and 220 underwent PFNA (PFNA group) internal fixation. Demographic data, operation time, hospitalization time, blood loss, intraoperative transfusion, complications, Harris hip scores (HHS), and Parker-Palmer mobility scores (PPMS) were compared between the two groups.

**Results:**

The two groups were comparable in terms of demographic data (*p* > 0.05). The operation time in the PFNA group was shorter than that in the PFUN group, and blood loss was less than that in the PFUN group (*p* < 0.05). However, the hospitalization time was shorter in the PFUN group than in the PFNA group (*p* < 0.05). At 3 and 6 months postoperatively, the HHSs of the PFUN group were significantly higher than those of the PFNA group, and similar results were obtained for PPMS at 6 months postoperatively (*p* < 0.05). Nevertheless, we found no difference between the groups in the HHSs and PPMSs at 12 months postoperatively or at the final follow-up (*p* > 0.05). The rate of fixation failure in the PFUN group was significantly lower than that in the other groups (*p* < 0.05), whereas there was no significant difference in the rate of other general complications (*p* > 0.05). Among patients with AO/OTA classification 31-A2, the implant failure rate in the PFUN group was significantly lower than that in the other group, and the PPMS score at 6 months postoperatively was higher (*p* < 0.05). In the AO/OTA classification of 31-A3 stratification, the Harris score and PPMS score of the PFUN group during the postoperative follow-up period were higher than those of the PFNA group (*p* < 0.05).

**Conclusion:**

The PFUN is an effective and safe internal fixation device for IFFs, especially for unstable medial and lateral wall fractures. Compared to PFNA, PFUN can significantly reduce the rate of implant failure in IFFs and accelerate postoperative rehabilitation. For IFFs with AO/OTA classifications of 31-A2 and A3, PFUN has a greater advantage than PFNA.

## Introduction

With an aging global population, the prevention and treatment of hip fractures have become a public health issue. Intertrochanteric femoral fractures (IFF) are the most common type of hip fractures, accounting for approximately 54% of all hip fractures (1, 2). For patients with IFF, it has become a consensus that surgical treatment should be actively arranged if they can tolerate anasthesia and surgery, due to the serious impact on their daily living abilities and various serious complications (3, 4). A meta-analysis of 36 RCT studies (5) showed that proximal femoral nail anti-rotation (PFNA) was significantly superior to the dynamic condylar screw, less invasive stabilization system, proximal femoral nail (PFN), and other internal fixation methods for reducing bleeding and restoring hip function. Therefore, PFNA has gradually become the mainstream intramedullary fixation method for IFF.

However, despite improved techniques and various implant modifications, implant failure remains a problem for IFF, especially unstable ones (6). Implant failure is a possible secondary operation that may increase the mortality rate in older people. Therefore, it was essential to reduce the implant failure rate in the treatment of IFF. Various factors have been reported to be related to implant failure in the IFF after intramedullary fixation (7, 8). The integrity of the medial and lateral femoral walls has been identified as a prognostic factor in the identification of unstable pertrochanteric fractures and in the prognosis of surgical treatment (7–9). Nonetheless, the existing intramedullary fixation system does not have a device to reconstruct the medial and lateral supports of the IFF separately or simultaneously.

Our team developed a new design for the cephalomedullary nail called the proximal femoral universal nail (PFUN) for the reconstruction of the medial and lateral support of the IFF, even for any type of proximal femoral fracture. This study aimed to compare the clinical outcomes of PFUN and PFNA internal fixation for the treatment of IFF in older patients.

## Methods

This retrospective study was complied with the principles of the Declaration of Helsinki and approved by the ethics committee of Peking University Third Hospital (Approval Number: IRB00006761-M2024582). And the study was retrospectively registered at ClinicalTrials.gov (NCT06277622) in Feb 23 2024. Written informed consent was obtained from each participant before commencement.

### Design and population

Between January 2022 and January 2024, 470 patients with IFF treated with closed reduction and intramedullary fixation were included in the data analysis. After applying the inclusion and exclusion criteria, 435 patients (92.6%) were included. Among them, 215 patients underwent PFUN (PFUN group) and 220 patients underwent PFNA (PFNA group). The inclusion criteria were (1) age ≥ 60 years; (2) unilateral IFF were confirmed using radiography, and the integrity of medial and lateral walls could be distinguished by computed tomography; (3) operative treatment of closed reduction and internal fixation was undergone by PFUN or PFNA; and (4) at least 12 months of follow-up. The exclusion criteria were as follows: (1) age < 60 years; (2) pathological or open fractures; (3) multiple traumas; (4) American Society of Anesthesiologists (ASA) score V; and (5) refusal to participate.

### Surgical strategy and postoperative care

All patients underwent intramedullary fixation performed by the same group of surgeons. After spinal anesthesia, surgical traction beds were used to fix patients in the supine position. During traction, the affected limb is internally rotated and placed in the traction frame. Closed reduction of the fractured leg was assisted by a G arm, which was routinely disinfected.

### PFUN

**Figure 1:**
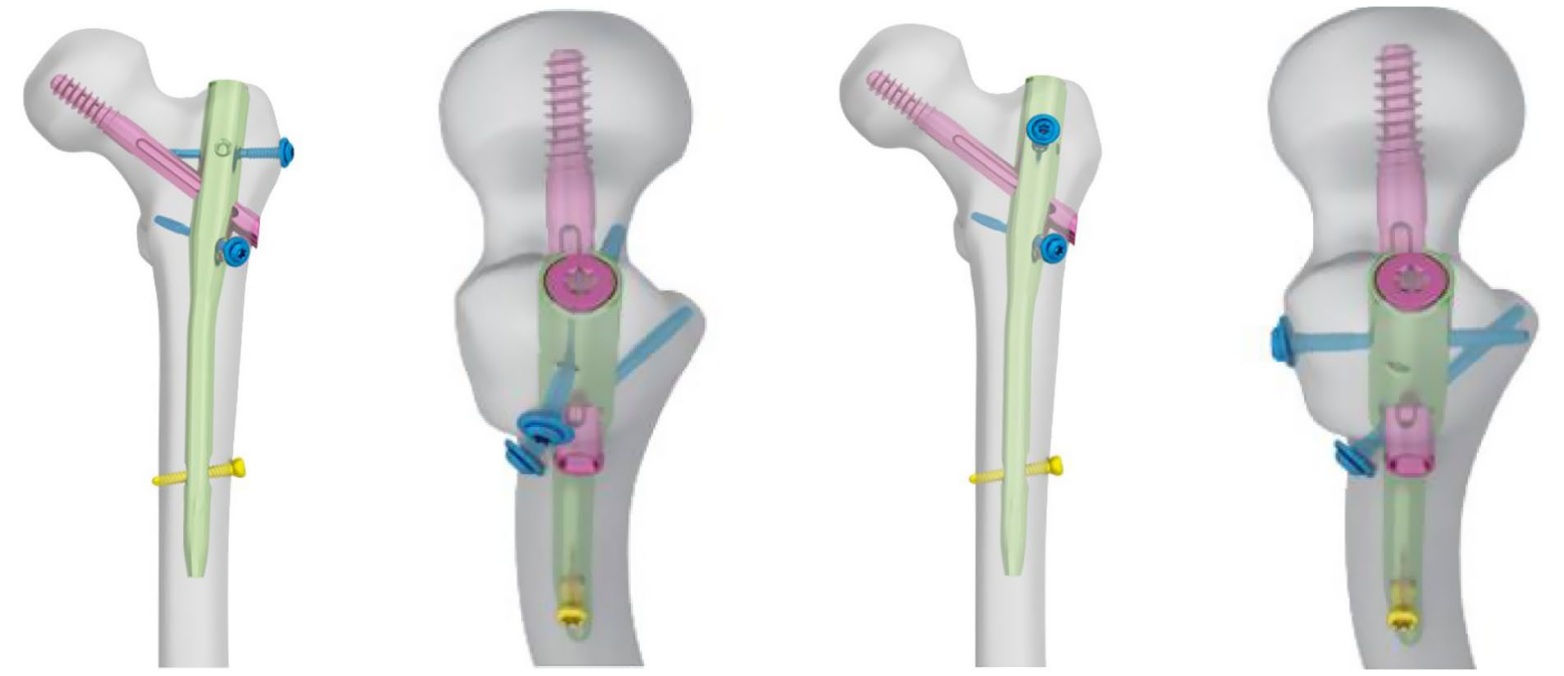
The schematic diagram of Proximal femoral universal nail (PFUN). PFUN was made up by the usual main nail, the lag screw (or spiral blade), the lock nail, the lesser trochanteric screw and a lateral wall screw(or a coronal screw).

In this internal fixation system, apart from the usual main nail, lag screw (or spiral blade), and lock nail, it consists of a lesser trochanteric screw to fix the lesser trochanteric fragment (the medial femoral wall fragment) and a lateral wall screw to fix the lateral femoral wall fragment (or a coronal screw to fix the coronal fracture fragment; Figure 1).

**Figure 2:**
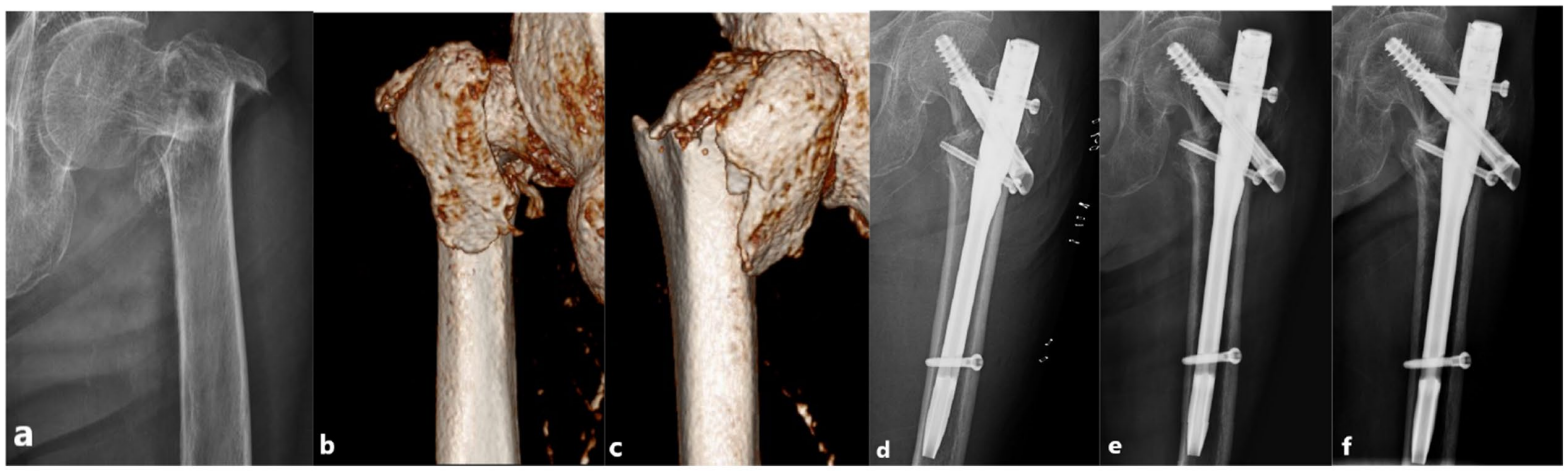
PFUN internal fixation in a 79-year-old female patient with a left intertrochanteric fracture. The patient experienced pain in the left hip and limited movement after the fall, and was unable to walk. At the last follow-up at 16 months after the operation, the Harris score was 89 points and the patient can walk independently. **(a)** X-ray image showing intertrochanteric fracture of the femur after the injury. **(b)** CT showing the fractures of the medial femoral wall. **(c)** CT showing the fractures of the lateral femoral wall. **(d)** X-ray image immediately after the operation showing appropriate fracture reduction. The lesser trochanteric fragment (the medial femoral wall fragment) was fixed with a lesser trochanteric screw and the lateral femoral wall fragment was fixed with a lateral wall screw. **(e)** X-ray image showing appropriate internal fixation evolution 3 months after the operation. **(f)** X-ray image showing appropriate fracture healing 12 months after the operation.

A 5 cm longitudinal incision was made outside the apex of the greater trochanter of the femur to expose the greater trochanter. After selecting the insertion point at which the apex of the greater trochanter deviated slightly from the inside, the surgeon used an opener to expand the bone-opening window and inserted a guide needle into the medullary cavity. After confirming the correct placement of the guide needle using G-arm fluoroscopy, the surgeon enlarged the entry point and inserted the main nail (Double Medical Technology Co., Ltd., Xiamen, China) into the appropriate position. Another guide needle was inserted through the incision in the lateral femur using the aiming arm. After adjusting the angle and position of the needle using G-am fluoroscopy, the surgeon measured the appropriate length of the lag screw or spiral blade. A lag screw or spiral blade was inserted under fluoroscopy to ensure that the screw or blade was embedded in the center of the femoral head, and then a distal locking screw was inserted. When there is a displaced fracture or a coronal split fracture on the lateral wall, a lateral wall screw or a coronal screw would be placed routinely. And when there is a single or comminuted lesser trochanteric fragment (medial femoral wall fragment) involving the large posterior cortex near the base of the lesser trochanter, in which the fracture line reaches or exceeds the midline of the posterior wall, a medial wall screw would be placed. If fragments were present in the medial or lateral wall of the proximal femur that needed to be fixed, the surgeon used a lesser trochanteric screw to fix the lesser trochanteric fragment (the medial femoral wall fragment) and a lateral wall screw (or a coronal screw) to fix the lateral femoral wall fragment with the guide pin and the aiming arm. When the lateral femoral wall was comminuted, a plate was used to fix the comminuted lateral wall fragment. Finally, the nut was screwed to the tail of the main nail as a sealing cap. After fluoroscopic confirmation of fracture reduction and satisfactory internal fixation, the area was sutured closed, and the incision was bound to complete the operation (Figure 2).

### PFNA rotation

**Figure 3:**
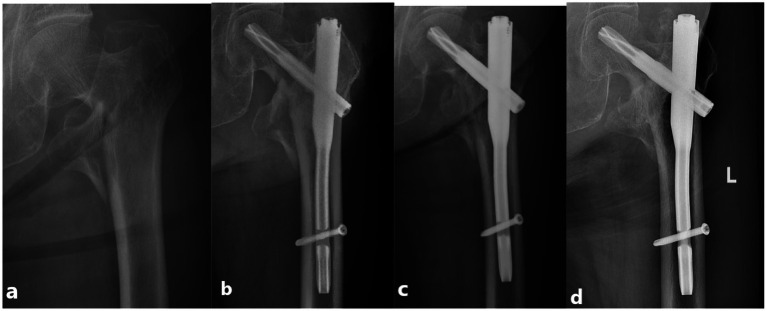
PFNA internal fixation in a 86-year-old male patient with a left intertrochanteric fracture. The patient experienced pain in the left hip and limited movement after the fall, and was unable to walk. At the last follow-up at 14 months after the operation, the Harris score was 85 points and the patient can walk independently. **(a)** X-ray image showing intertrochanteric fracture of the femur after the injury. **(b)** Postoperative X-ray showing appropriate fracture reduction. **(c)** X-ray image showing appropriate internal fixation position evolution 3 months after the operation. **(d)** X-ray image showing appropriate fracture healing 12 months after the operation.

The surgeon performed a 5 cm longitudinal incision at the lateral side of the apex of the greater trochanter of the femur, exposed the greater trochanter, and selected an insertion point slightly medial to the apex of the greater trochanter. A curved opener is used to open the apex of the greater trochanter. The guide pin was inserted into the center of the medullary cavity in the positive lateral position under G-arm fluoroscopy. The proximal end of the guide pin was used to define reaming. The main nail (Hebei Ruihe Medical Instrument Co., Ltd., Shijiazhuang, China) was inserted into the proper position, and the proximal aiming arm was used to insert the guide pin into the femoral neck. The position of the guide pin was verified using G-arm fluoroscopy. The length was then measured. The spiral blade was inserted toward the guide pin, which was positioned in the middle of the femoral head and tibia (as confirmed by fluoroscopy) and locked. The distal locking screw was then locked under the guidance of the aiming arm, and the main nail cap was screwed. Fracture reduction and internal fixation were confirmed using fluoroscopy, and the incision was washed, sutured, and bound to complete the operation (Figure 3).

Postoperative care was the same in both groups. Radiographs of the injured hips were also obtained after the surgical operation to evaluate reduction and fixation. All patients received prophylactic anti-infective treatment for 24 h after surgery. Passive motion is used to restore the muscle strength and joint function. All patients underwent radiological examinations at 1, 2, 3, 6, and 12 months postoperatively, and at the final follow-up. Full weight bearing was permitted only once the fracture line became obscured.

### Outcome measurement

Demographic data were recorded for both groups, including age, sex, body mass index (BMI), injury type, injury side, AO classification, ASA class, and comorbidities. The operation time, preoperation time (POT, the time from initial injury to surgery), hospitalization time, tip-apex distance (TAD) (10), neck-shaft angle (NSA) after the operation, quality of reduction, blood loss, and intraoperative transfusion (IT) between the two groups were assessed in this study. The quality of the postoperative reduction was graded as good, acceptable, or poor (11). It was good that both the forward and lateral displacements after reduction were less than one cortical thickness. It was acceptable if the anteroposterior or lateral displacement was less than one cortex. Otherwise, the reduction was graded as poor.

The primary outcome was the rate of fixation failure, including nonunion, implant cut-out, implant breakage, and implant back-out. Nonunion was defined as the state in which disturbed consolidation of a fracture that needs further surgical intervention or a prolonged healing time of more than 12 months. The secondary outcomes measured were the Harris hip score (HHS) evaluated at 3 months, 6 months, and 1 year postoperatively and the final follow-up, and the Parker-Palmer mobility score (PPMS) evaluated at 6 months, 1 year postoperatively, and the final follow-up. The HHS (12), a commonly used evaluation system for surgical results after hip surgery, includes a 100-point scale assessing pain, function, activity, deformity, and motion. The grade standard comprised three levels: 70 and below indicated a poor grade, 70–89 revealed fair/good quality, and 90 and above indicated an excellent grade. The PPMS (13) is used as the standard for evaluating the function of the affected limb in patients. Scores of 5 and below were considered poor, 6–7 were moderate, and 8–9 were excellent. Other general complications such as coxavara, wound infections, deep vein thrombosis, pneumonia, and urinary tract infections were also documented.

### Data analysis

We used SPSS v26.0 (SPSS, Chicago, IL, United States) for all statistical analyses. Categorical variables were compared using Fisher’s exact test or the chi-square test. Continuous variables with normal distribution were expressed as means±standard deviation and compared using Student’s t-test, and non-normally distributed variables were analyzed using the Mann–Whitney U test. Binary multivariate logistic regression analysis was used to correct confounding factors. The significance threshold was set at *p* < 0 0.05.

## Results

We analyzed the records of 435 patients: The PFUN group included 53 men and 162 women aged 60 to 100 years (average 80.4 ± 8.4 years). The injury type included high energy (1.4% or 3 of 215) and low energy (98.6% or 212 of 215), and the injury site was the right one in 96 cases and the left side in 119 cases. The PFNA group included 71 men and 149 women aged 60 to 99 years (average 79.5 ± 9.3 years). The injury type included high-energy (0.9%, 2 of 220) and low-energy (99.1%, 218 of 220) injuries, and the injury site was the right in 114 cases and the left in 106 cases. The two groups were comparable in terms of patient age, sex, BMI, injury type, injury side, AO/OTA classification, ASA score, and comorbidities (*p* > 0.05; Table 1). We found similar values for POT, TAD, and NSA after the operation, quality of reduction, and IT between the two groups (*p* > 0.05; Tables 1, 2). The operation time in the PFNA group was shorter than that in the PFUN group, and blood loss was less than that in the PFUN group (*p* < 0.05). However, the hospitalization time was shorter in the PFUN group than in the PFNA group (*p* < 0.05). At 3 and 6 months postoperatively, the HHSs of the PFUN group were significantly higher than those of the PFNA group, and similar results were obtained for PPMS at 6 months postoperatively (*p* < 0.05). The rate of fixation failure in the PFUN group was significantly lower than that in the other groups (*p* < 0.05), whereas, there was no significant difference in the rate of other general complications (*p* > 0.05; Table 2). All nine patients with fixation failure underwent reoperation and recovered at the final follow-up. The two patients with wound infections recovered after regular dressing changes. In cases with AO/OTA classification of 31-A1, there was no significant difference in the rate of fixation failure and clinical functional scores between the two groups (*p* > 0.05). Among patients with AO/OTA classification 31-A2, the rate of fixation failure in the PFUN group was significantly lower than that in the other group, and the PPMS score at 6 months postoperatively was higher (*p* < 0.05). In the AO/OTA classification of 31-A3 stratification, there was no significant difference in the fixation failure rate between the two groups (*p* > 0.05). However, the Harris score and PPMS score of the PFUN group during the postoperative follow-up period were higher than those of the PFNA group (*p* < 0.05; Table 3).

**Table 1:** Comparison of the patient characteristics of the PFUN group and PFNA group.

General information	PFUN group	PFNA group	*p* value
	*N* = 215	*N* = 220	
Age (years)	80.4 ± 8.4	79.5 ± 9.3	0.444
Sex			
Male	53	71	0.078
Female	162	149	
BMI	23.7 ± 4.1	23.4 ± 4.0	0.337
Injury type			0.682
High energy	3	2	
Low energy	212	218	
Injury side			0.460
Left	119	114	
Right	96	106	
AO/OTA classification			0.081
31-A1	44	64	
31-A2	157	139	
31-A3	14	17	
ASA score			0.488
I	8	11	
II	157	158	
III	50	49	
IV	0	2	
Comorbidities			
Hypertension	131	122	0.247
Diabetes	62	69	0.566
Coronary heart disease	41	37	0.541
Cerebral infarction	38	34	0.533
Dementia	5	9	0.297
Quality of reduction			0.300
Good	152	145	
Acceptable	57	63	
Poor	6	12	
TAD			0.405
>25 mm	28	23	
<25 mm	187	197	
NSA after the operation (°)	137.8 ± 77.2	132.6 ± 5.2	0.936
POT(days)	4.0 ± 3.2	4.5 ± 3.6	0.330

Data represent the mean [SD]; BMI, body mass index; ASA, American Society of Anesthesiologists; TAD, tip-apex distance; NSA, neck-shaft angle; POT: preoperation time; PFUN, proximal femoral universal nail; PFNA, proximal femoral nail anti-rotation.

**Table 2:** Perioperative and postoperative follow-up data.

	PFUN group	PFNA group	*p* value
	*N* = 215	*N* = 220	
Hospitalization time (days)	4.0 ± 1.9	4.9 ± 5.7	0.002
Operation time (min)	67.8 ± 28.1	61.1 ± 23.8	0.005
Blood loss (ml)	87.3 ± 117.0	68.3 ± 69.4	0.031
IT	34(15.8%)	40(18.2%)	0.511
Follow-up time (m)	16.1 ± 2.7	15.9 ± 3.4	0.148
HHS			
3 months	68.3 ± 3.8	67.0 ± 5.2	0.023
6 months	76.9 ± 3.9	75.4 ± 5.0	0.003
12 months	85.7 ± 2.7	84.9 ± 4.3	0.540
Final follow-up	88.9 ± 2.6	88.1 ± 4.0	0.127
PPMS			
6 months	5.7 ± 0.6	5.5 ± 0.6	<0.001
12 months	6.9 ± 0.5	6.8 ± 0.6	0.218
Final follow-up	7.5 ± 0.7	7.5 ± 0.7	0.375
Fixation failure	1(0.5%)	8(3.6%)	0.037
Non-union	0	2	
Cut-out	1	3	
Breakage	0	1	
Back-out	0	2	
Wound infections	2(0.9%)	0	0.244
Deep vein thrombosis	14(6.5%)	15(6.9%)	0.898
Pneumonia	7(3.3%)	9(4.1%)	0.644
Urinary tract infection	10(4.7%)	4(1.8%)	0.094

Data represent mean [SD]; IT, intraoperative transfusion; HHS, Harris Hip Score; PPMS, Parker-Palmer mobility score; PFUN, proximal femoral universal nail; PFNA, proximal femoral nail anti-rotation.

**Table 3:** The primary and secondary outcomes of stratified comparison according to AO/OTA classification.

AO/OTA classification	31-A1			31-A2			31-A3		
	PFUN	PFNA	*p* value	PFUN	PFNA	*p* value	PFUN	PFNA	*p* value
	*N* = 44	*N* = 64		*N* = 157	*N* = 139		*N* = 14	*N* = 17	
HHS									
3 months	69.3 ± 2.6	69.1 ± 5.3	0.463	68.2 ± 3.8	67.1 ± 4.9	0.114	66.4 ± 5.8	57.2 ± 4.1	<0.001
6 months	77.6 ± 3.2	77.3 ± 2.8	0.357	76.8 ± 3.9	75.7 ± 4.7	0.051	75.1 ± 6.0	66.1 ± 3.9	<0.001
12 months	86.3 ± 1.7	86.6 ± 1.4	0.213	85.7 ± 2.6	84.8 ± 4.2	0.385	84.5 ± 5.6	79.3 ± 6.6	0.006
Final follow-up	89.1 ± 1.7	89.6 ± 1.7	0.195	88.9 ± 2.5	88.0 ± 3.7	0.055	88.1 ± 5.3	83.3 ± 7.3	0.007
PPMS									
6 months	5.8 ± 0.5	5.6 ± 0.5	0.074	5.7 ± 0.6	5.5 ± 0.6	0.001	5.4 ± 0.7	4.8 ± 5.1	0.044
12 months	7.0 ± 0.4	7.0 ± 0.3	0.594	6.8 ± 0.5	6.8 ± 0.5	0.492	6.7 ± 0.8	5.9 ± 1.1	0.036
Final follow-up	7.6 ± 0.5	7.7 ± 0.5	0.506	7.5 ± 0.7	7.5 ± 0.6	0.402	7.5 ± 0.9	6.7 ± 1.2	0.048
Fixation failure	0	0	-	0	4(2.9%)	0.048	1(7%)	4(23.5%)	0.344
Non-union	0	0		0	0		0	2(11.8%)	
Cut-out	0	0		0	2(1.4%)		1(7%)	1(5.9%)	
Breakage	0	0		0	1(0.7%)		0	0	
Back-out	0	0		0	1(0.7%)		0	1(5.9%)	

Data represent mean [SD]; HHS, Harris Hip Score; PPMS, Parker-Palmer mobility score; PFUN, proximal femoral universal nail; PFNA, proximal femoral nail anti-rotation.

A binary multivariate logistic regression model for correcting confounding factors was fitted with whether the internal fixation failed as the dependent variable, the group as the independent variable, and AO/OTA classification and comorbidities as confounding factors. The differences before and after correction were both statistically significant (Table 4).

**Table 4:** Comparison of the internal fixation failure rates between the two groups.

	B	Standard error	Wald	Odds ratio (95%CI)	*p* value
Before correcting confounding factors					
PFUN Group					1.00 (reference)
PFNA Group	2.211	1.059	4.364	9.128 (1.146 ~ 72.679)	0.037
After correcting confounding factors					
PFUN Group					1.00 (reference)
PFNA Group	2.851	1.202	5.624	17.310 (1.640 ~ 182.670)	0.018

PFUN, proximal femoral universal nail; PFNA, proximal femoral nail anti-rotation.

## Discussion

IFF accounts for over 50% of hip fractures in the older population, and over 50% of these fractures are unstable (14). The objective of treating IFFs is to obtain strong fixation, rebuild the anatomic continuity and mechanical stability of the proximal femur, reduce the incidence of complications related to long-term bed rest, restore lower limb function as soon as possible, and ultimately improve the quality of life of patients (4). Compared with extramedullary fixation, intramedullary nails are more popular because of their shorter operation time, reduced blood loss, biomechanical stability, and improved walking ability in unstable IFFs (15, 16). However, secondary lateral wall fractures and complications related to implants, such as screw back-out, breakage, and cut-out, still occur with the use of intramedullary nails, particularly for unstable fractures (17). Hu et al. (11) reported that it was difficult for intramedullary nailing to avoid reinjury of the lateral wall, particularly for severe comminuted fractures. Various factors have been reported to be related to the occurrence of implant failure for IFF after intramedullary fixation (7–9), and the stability of fractures after internal fixation is very important.

The proximal part of the femur is mainly affected by compressive stress and tensile stress. When the femur is bearing weight, the medial wall of the proximal part mainly bears compressive stress, while the lateral cortical bone provides support for tensile stress. In our previous studies (8, 9), we found that the integrity of the lateral femoral wall associated with the prognosis of proximal femoral fractures. The lateral wall of the proximal femur is an important structure for the stability of IFF, providing lateral support for the proximal fragment. If patients with proximal femoral fractures combined with lateral femoral wall fractures are to receive effective treatment, lateral femoral wall reconstruction is necessary. In another previous study by our group (7), we reported that a single or comminuted lesser trochanteric fragment (medial femoral wall fragment) involving the large posterior cortex near the base of the lesser trochanter, in which the fracture line reaches or exceeds the midline of the posterior wall, had a significantly increased failure rate. It was significant to identify this kind of fragment when conducting preoperative planning, and reduction of the medial wall fragment and fixation should be considered during surgery using intramedullary nails. In the PFNA group, all eight IFFs with fixation failure were combined with medial or lateral wall fractures that required fixation. In a meta-analysis, Lee et al. (18) reported that the failure rate of intramedullary fixation for unstable IFFs was 0–18.2%. To our knowledge, none of the intramedullary nails currently used in the clinic can independently solve the problem of medial and lateral femoral wall fractures and enhance stability, resulting in a high rate of internal fixation failure. In the proximal femoral universal nail system, the lateral wall screw and lesser trochanteric screws can fix the lateral and medial femoral wall fragments to reconstruct the compressive stress support and tensile support of the proximal femur, thereby increasing the success rate of internal fixation. In our initial biomechanical studies about PFUN (19, 20), a larger axial stiffness, higher average torque and higher failure load were found in the PFUN when compared with the PFNA for proximal femoral fractures with broken medial wall and lateral wall. And the finite element analysis results showed that the PFUN model had a higher stress concentration compared with the PFNA model, and the total displacement of the PFNA model increased by 11.63% when compared with the PFUN model in the proximal femoral fracture with broken medial wall and lateral wall. And then, in this study, the PFUN group had a significantly lower fixation failure rate than the PFNA group. Our results showed that PFUN achieved relatively good clinical results, which was consistent with our expectations.

The functional scores and perioperative data of the two groups demonstrated the advantages of the two internal fixation methods. The HHS was higher in the PFUN group than in the PFNA group at 3 and 6 months postoperatively. Similar results were obtained for PPMS 6 months postoperatively. This is probably because the patients in the two groups differed when full weight-bearing and full activity began, which was dependent on when the fracture line became obscured on radiological imaging. In a biomechanical study (21), fixation with PFNA and a cerclage wire for the IFF with a lesser trochanteric fragment reduced the movement of the femoral neck and increased the overall stiffness significantly. In the PFUN group, the medial or lateral wall fragments were fixed to recover the mechanical characteristics of the proximal femur and achieve more stable internal fixation; thus, the patients in the PFUN group began rehabilitation exercises earlier. At long-term follow-up, no statistically significant differences were noted in HHS and PPMS between the two groups, indicating that the patients in the two groups achieved similar functional recovery in the later stage. The operative time in the PFNA group was shorter, and the wound infection rate in the PFNA group was numerically lower than that in the PFUN group; however, the difference was not statistically significant between the two groups. Shortening the operative time may help reduce the impact of anesthesia and surgery on patients and the incidence of infection. Blood loss was lower in the PFNA group than in the PFUN group. Increased blood loss may prolong the time required for postoperative recovery. However, the PFUN group did not have a significantly higher proportion of intraoperative transfusions or a longer hospitalization time than the other groups. For older patients with comorbidities, prolonged operation time and increased blood loss may increase the perioperative risk. Accordingly, we believe that PFUN may not be suitable for patients with poor physical conditions and low surgical tolerance. However, further studies are required to confirm this hypothesis.

In the stratified comparison according to AO/OTA classification, The advantages of PFUN are mainly reflected in cases of type 31-A2 and A3 fractures. Among patients classified as 31-A2, the implant failure rate in the PFUN group was significantly lower. In cases classified as 31-A3, even though there was no significant difference in the statistical results, the fixation failure rate in the PFNA group was numerically higher than that in the PFUN group. Therefore, we initially believe that PFUN has more advantages in treating IFFs with AO/OTA classification 31-A2 and A3.

This study has certain limitations. First, this was a large single-center retrospective cohort study, which may have resulted in selection bias. Therefore, multicentre prospective randomized controlled trials are required to verify our conclusions. Second, we lacked information on the long-term status of patients after surgery, and studies with extended follow-up periods are required. Third, the clinical scores in this study, such as the HHS and PPMS, had subjective components that may have led to errors. Therefore, more objective and high-quality questionnaires are required to evaluate patient recovery after IFF. Finally, other factors that may affect fracture stability, such as bone mineral density and the position of internal fixation, were not included. Subsequent studies should conduct a more comprehensive assessment of patients.

## Conclusion

PFUN is an effective and safe internal fixation device for IFFs, particularly for unstable medial or lateral wall fractures. Compared to PFNA, PFUN can significantly reduce the rate of implant failure in IFFs and accelerate postoperative rehabilitation. For IFFs with AO/OTA classifications of 31-A2 and A3, PFUN has a greater advantage than PFNA.

## Data Availability

The original contributions presented in the study are included in the article/supplementary material, further inquiries can be directed to the corresponding authors.
